# Entrapment of Cyanase from *Thermomyces lanuginosus* Using Biomimetic Silica and Its Application for Cyanate Bioremediation

**DOI:** 10.3390/polym16182594

**Published:** 2024-09-13

**Authors:** Su-Chun How, Chia-Jung Hsieh, Chi-Yang Yu

**Affiliations:** Department of Chemical Engineering and Biotechnology, Tatung University, No. 40, Sec. 3, Zhongshan N. Rd., Taipei 104, Taiwan; schow@gm.ttu.edu.tw (S.-C.H.); cc1235896te@gmail.com (C.-J.H.)

**Keywords:** biomimetic silica, cyanase, carbonic anhydrase, cyanate, entrapment

## Abstract

Cyanate, a toxic product from the chemical oxidation treatment of highly toxic cyanide, can be converted to harmless ammonia and carbon dioxide by cyanase (EC 4.2.1.104). Cyanase from *Thermomyces lanuginosus* was entrapped in biomimetic silica to improve stability and reusability. After entrapment, the enzyme’s activity increased by two-fold, and the residual activity after 30-min of incubation at 60 °C also increased by two-fold, compared to the free enzyme. After being stored at room temperature for 28 days, the entrapped cyanase retained 79% of the initial activity, while the free form retained 61%. The immobilized cyanase was successfully applied to cyanate detoxification; the co-entrapment of carbonic anhydrase from *Sulfurihydrogenibium azorense* decreased the amount of bicarbonate necessary for cyanate detoxification by 50%. The cyanate degradation retained 53% of the initial value after the co-entrapped cyanate and carbonic anhydrase were reused five times.

## 1. Introduction

Cyanase (EC 4.2.1.104), using HCO_3_^−^ as a substrate, converts cyanate to NH_3_ and CO_2_. Cyanase is found in bacteria, fungi, and plants [1,2,3]; its physiological roles include nitrogen assimilation and cyanate detoxification. Cyanate can serve as the only energy source and reductant for certain organisms by expressing cyanase upon the addition of cyanate [1]. One important industrial application of cyanase is the bioremediation of the toxic cyanate [2,3], an oxidized derivative of the highly poisonous cyanide. Cyanides are widely used in industry and have a variety of applications, such as the extraction of precious metals, electroplating, and the synthesis of organic chemicals; the annual worldwide consumption of cyanide is over 1.5 million metric tons [4]. The existing treatments for cyanide-containing waste are effective; however, they are expensive and utilize reagents that are hazardous to the environment [5]. The bioremediation of cyanide can be an ecologically viable alternative to the existing treatments.

Cyanase originated from *Thermomyces lanuginosus* (TlCyn), a thermophilic fungus that has very high activity, and the activity is not affected by the presence of metal ions up to 5 mM [6]. It also has good thermostability and pH stability; these unique properties render TlCyn an ideal choice for cyanate bioremediation under extreme environmental conditions. In a lab-scale experiment, TlCyn could degrade 90% of the 10 mM cyanate present in the wastewater [6].

For a large-scale enzymatic process, such as the bioremediation of cyanate, the biocatalyst’s cost often determines the process’s economic feasibility. One common approach to reduce the cost of the enzyme is to perform immobilization. Although cyanase has important industrial applications, the immobilization of cyanase has yet to be explored. To the best of our knowledge, the only reported immobilization supports for cyanase are glass beads [7], magnetic nanoparticles [8], and magnetic multiwall carbon nanotubes [9]; covalent bonds were formed between the cyanases and the supports.

The results from previous investigations of the main conventional enzyme immobilization methods, including adsorption [10], entrapment [11], microencapsulation [12], covalent binding [13], and cross-linking [14], have been reported. These methods have been widely studied and applied for immobilizing various enzymes on different support materials. However, they have certain limitations that hinder their development. These limitations include the harsh conditions often required, such as extreme pH, high temperatures, and the use of organic solvents, which can denature enzymes. Additionally, these methods typically have lower enzyme loading capacities, usually limited to 0.1–5% (w/w). Furthermore, significant enzyme activity loss upon immobilization is common, with decreases of 70–90% often reported [15]. Compared to conventional enzyme immobilization methods, biomimetic silica has several advantages: (1) mild reaction conditions, (2) short reaction time, (3) high immobilization efficiency, (4) low leakage, and (5) ease of operation [16,17]. Biosilica serves as the structural skeleton for marine organisms, such as diatoms, radiolarians, and siliceous sponges; it is also used in higher plants, such as rice and cucumber, to protect against stresses [18]. One well-studied example of biosilicification is *Cylindrotheca fusiformis*, a marine diatom; highly post-translationally modified proteins named silaffins serve as templates for forming siliceous cell walls [19]. Cationic macromolecules, such as poly-L-arginine, polyethyleneimine, and polyallylamine, can also promote the formation of biomimetic silica [20].

To improve the stability and reusability of TlCyn, we used polyallylamine as a cationic template to entrap TlCyn in biomimetic silica. The protonated amine functional groups of polyallylamine catalyzed the condensation of silica from silicic acid; the polyallylamine molecules also facilitated silica formation by serving as templates/scaffolds [20]. The biochemical properties of free and entrapped TlCyn were determined and compared; the performance of cyanate detoxification using entrapped TlCyn was also examined. In addition, the feasibility of the co-entrapment of TlCyn and carbonic anhydrase (EC 4.2.1.1) for improving the cyanate detoxification was demonstrated.

## 2. Materials and Methods

### 2.1. Bacterial Strains and Vector Construction

For this study, we employed a cloning method previously established in literature, using *E. coli* DH5α for cloning and *E. coli* BL21(DE3) for recombinant protein expression [21]. Following the approach from an earlier work, the TlCyn gene—corresponding to the amino acid sequence listed under entry code 6XGT_A in the Protein Data Bank—was commercially synthesized. The gene was then inserted between the NdeI and XhoI restriction sites of a pET-28a(+) vector. Consistent with the established protocol, a stop codon was introduced before the XhoI site to ensure the production of a recombinant TlCyn protein with an N-terminal (His_6_)-tag.

### 2.2. Expression and Purification of TlCyn

The expression and purification procedures followed the methods outlined in a previous study [2], with slight modifications. *E. coli* BL21(DE3) cells transformed with the pET-28a(+)-TlCyn plasmid were cultured in LB medium containing 50 µg/mL kanamycin at 37 °C. Once the culture reached OD_600_ of 0.5 and 0.7, Tlcyn expression was induced with 0.8 mM IPTG. The culture was then incubated for an additional 8 h at 25 °C before harvesting the cells. The cell pellet was lysed at room temperature for 20 min using the BugBuster protein extraction reagent (Merck, Taiwan), with the addition of Benzonase Nuclease and rLysozyme (Sigma-Aldrich, USA), as per the manufacturer’s protocol. Purification of TlCyn was carried out using the Ni-NTA His-Bind Resin Chromatography kit (Merck). The purified enzyme was then desalted into a storage buffer of 50 mM Tris-HCl (pH 8), and its concentration was determined via the Bradford assay using BSA as a standard.

### 2.3. Activity Assays

The cyanase activity assay was based on the method reported by Ranjan et al. [6], with some modifications. To begin, 0.5 mL of 50 mM Tris-HCl (pH 8) was mixed with 0.2 mL of 10 mM KOCN and 0.2 mL of 15 mM NaHCO_3_ in a microcentrifuge tube. The reaction was initiated by introducing 0.1 mL of cyanase at a concentration of 3 µg/mL, followed by a 10 min incubation at 60 °C. To halt the reaction, 1 mL of Nessler’s reagent was added, and the absorbance at the characteristic wavelength of 420 nm was recorded using a UV-visible spectrophotometer (JASCO V-550, Japan) at room temperature. The amount of ammonium formed was quantified against a calibration curve constructed from NH_4_Cl standards. One unit (U) of cyanase activity corresponded to the production of 1 μmol of ammonium per minute. The hydratase activity of CA was measured using a method detailed in a separate study [22] and was reported in Wilbur–Anderson units (WAU), defined as

(1) $$WAU=T_{0}-T/T$$

where *T*_0_ and *T* are the times for the pH to decrease from 8.3 to 6.3 in uncatalyzed and catalyzed reactions, respectively. All assays and tests were performed in triplicate.

### 2.4. Entrapment of TlCyn

One millimolar silicic acid was prepared by mixing 152 µL of tetramethyl orthosilicate with 848 µL of 1 mM HCl; the mixture was incubated at room temperature for 15 min under rotary shaking at 50 rpm. To entrap the enzyme, 1 mL of 2 mM polyallylamine hydrochloride in D.I. water, 8 mL of 2.4 µg/mL TlCyn in the storage buffer, and 1 mL of freshly prepared silicic acid were mixed in a 15 mL centrifuge tube; 5 mL of 0.1 M KH_2_PO_4_ (adjusted to pH 8.0 with 0.1 N NaOH) was added to the mixture to initiate the silicification reaction. After incubating for 5 min at room temperature, the suspension was centrifuged at 6000× *g* for 5 min, and then the supernatant was removed. To wash the silica particles containing TlCyn (TlCyn-SP), the particles were resuspended with 10 mL of the storage buffer, followed by centrifugation at 6000× *g* for 5 min, and then the supernatant was removed; the process was repeated once more. Finally, the washed TlCyn-SP was resuspended in 15 mL of the storage buffer and stored under refrigeration for future use.

### 2.5. Cyanate Degradation by TlCyn-SP

In a microcentrifuge tube, 0.5 mL of 50 mM Tris-HCl (pH 8.0), 0.2 mL of 20 mM KOCN, and 0.2 mL of NaHCO_3_ at varying concentrations (2.5, 5, 7.5, 10, 12.5, and 15 mM) were mixed thoroughly using a vortex. The mixture was then placed in a water bath at 60 °C, where 235 µL of TlCyn-SP was added. The sample was incubated for 10 min while being shaken at 100 rpm. Ammonium released from the cyanate degradation was quantified following the procedure outlined in Section 2.3. To assess the impact of the matrix on degradation, 50 mM Tris-HCl buffer was replaced with industrial wastewater collected from the electric motor factory of the Tatung Company in New Taipei City, Taiwan, and the metal ion content of the wastewater was determined using an inductively coupled plasma-optical emission spectrometer (ICP-OES), specifically the Perkin Elmer Optima-2000 DV, USA.

## 3. Results and Discussion

### 3.1. Expression and Purification of TlCyn

**Figure 1:**
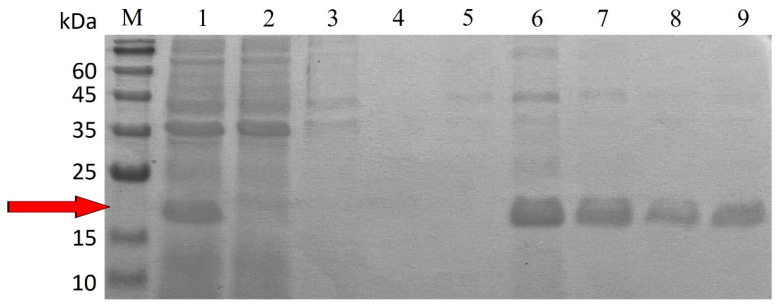
The SDS-PAGE image of the purification of TlCyn. Lane M: marker; Lane 1: lysate; Lane 2: flow-through; Lanes 3 and 4: washing fractions; Lanes 5–8: eluted fractions; Lane 9: desalted TlCyn. The red arrow indicates the position corresponding to the molecular weight of TlCyn (20,085 Da).

The expression levels post-induction were monitored at 2 h intervals using SDS-PAGE, revealing that a plateau was achieved at 8 h. Consequently, the 8 h post-induction incubation was chosen for further experiments. The recombinant TlCyn consisted of 181 amino acids and had a theoretical molecular weight of 20,085 Da, which is consistent with the results observed on SDS-PAGE (Figure 1). Affinity column purification resulted in a purity of 98%, as determined with densitometry. TlCyn had an average specific activity of 7.3 ± 0.1 × 10^4^ U/mg (N = 3), which is slightly lower than that reported by Ranjan et al. [6]. The yield was about 16 mg of recombinant TlCyn per liter of culture.

### 3.2. Entrapment of TlCyn

The entrapment efficiency and activity recovery were examined using 8 mL of 2.4, 6, 20, 60, and 200 μg/mL of TlCyn in the reaction. The entrapment efficiency is defined as the amount of the entrapped enzyme divided by that of the added enzyme; the activity recovery is defined as the specific activity of the entrapped enzyme divided by that of the free enzyme. The entrapment efficiency was close to 100% for all the enzyme concentrations tested because no cyanase activity was found in the supernatant and the washing fractions, showing that most of the TlCyn molecules were immobilized inside the silica. The high entrapment efficiency agrees with our previous work [22,23], which entrapped CA. The activity recovery ranged from 200% to 243% for all the enzyme concentrations tested, indicating that the activity increased after immobilization. The enhanced activity of immobilized TlCyn could be related to the improved thermal stability at the assay temperature of 60 °C, which is discussed in Section 3.3. The highest activity recovery of 243% was observed at 2.4 μg/mL of TlCyn, and this concentration was used for later entrapment. An SEM image of TlCyn-SP revealed that most of the silica particles formed an agglomerate; the average diameter was 29 ± 2.6 nm (N = 10).

### 3.3. Effect of Temperature on Activity and Stability

**Figure 2:**
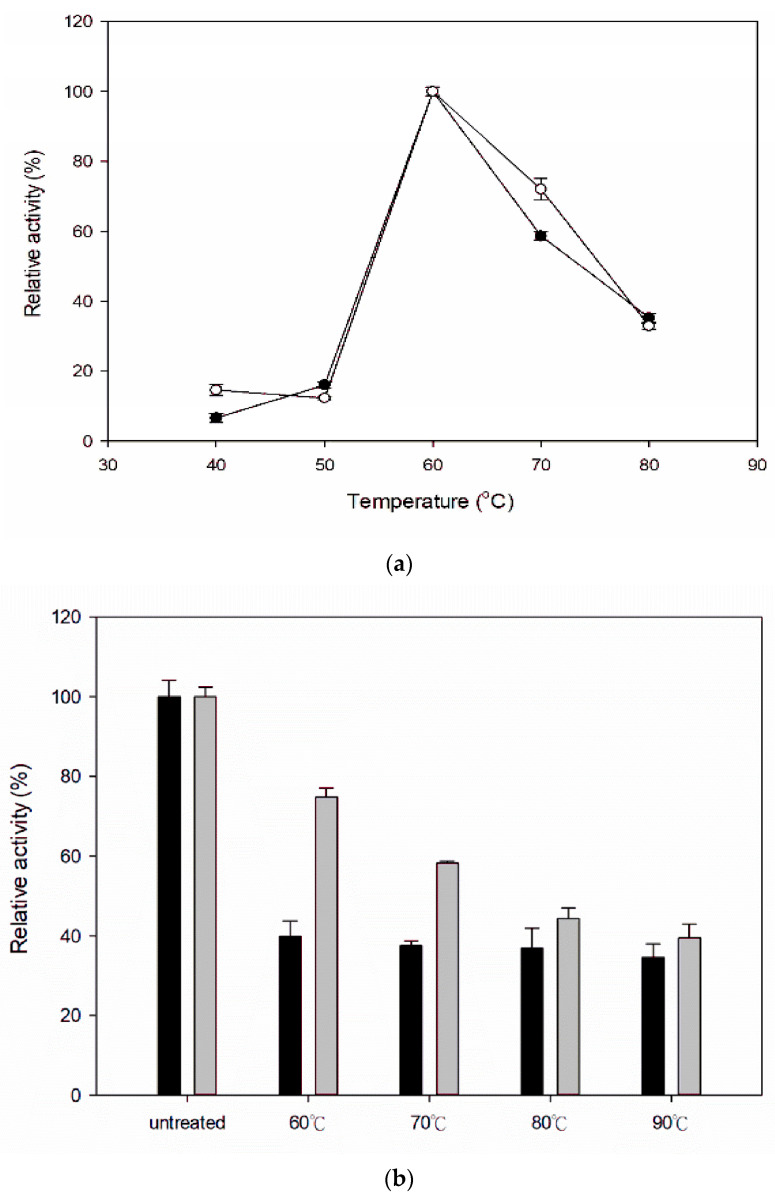
The effect of temperature on (**a**) activity and (**b**) stability. Solid circles: TlCyn; open circles: TlCyn-SP (**a**). Black bars: TlCyn; gray bars: TlCyn-SP (**b**). The stability was examined by incubating the enzyme under a designated temperature for 30 min before assaying its activity. The activities of untreated TlCyn and TlCyn-SP were set as 100%.

The effect on activity is shown in Figure 2a. At 40 and 50 °C, TlCyn exhibited less than 17% of its highest activity, which was observed at 60 °C. The activity decreased significantly when the temperature was further increased to 70 °C and 80 °C; only one-third of the highest activity was retained at 80 °C. The entrapment did not alter the effect of temperature on activity because the activity–temperature profile of TlCyn-SP is similar to that of TlCyn. The optimal temperature for both forms of TlCyn was 60 °C, the same as a previous report [6].

The effect on stability was examined by incubating the enzyme under a designated temperature for 30 min before assaying its activity, and the results are shown in Figure 2b. The free TlCyn exhibited a significant decrease in activity; the remaining activity was in the range of 35% to 40%, regardless of the testing temperature. The biomimetic silica clearly improved the thermal stability; at 60 °C and 70 °C, the residual activities increased from 40% and 38% to 75% and 58%, respectively. The residual activity of TlCyn-SP was almost two-fold higher than that of TlCyn at 60 °C, which partly explains the increase in activity after immobilization described in Section 3.2. However, the improvement in thermal stability was less evident at 80 °C and 90 °C. It has been shown that the quaternary structure of a decamer is likely necessary for TlCyn to be active [24]; improved thermal stability could result from the prevention of the dissociation of the decamer by the silica network. Improved thermal stability could also be related to a structurally more rigid subunit because the movement of the enzyme molecule is constrained in the silica matrix.

### 3.4. Effect of pH on Activity and Stability

**Figure 3:**
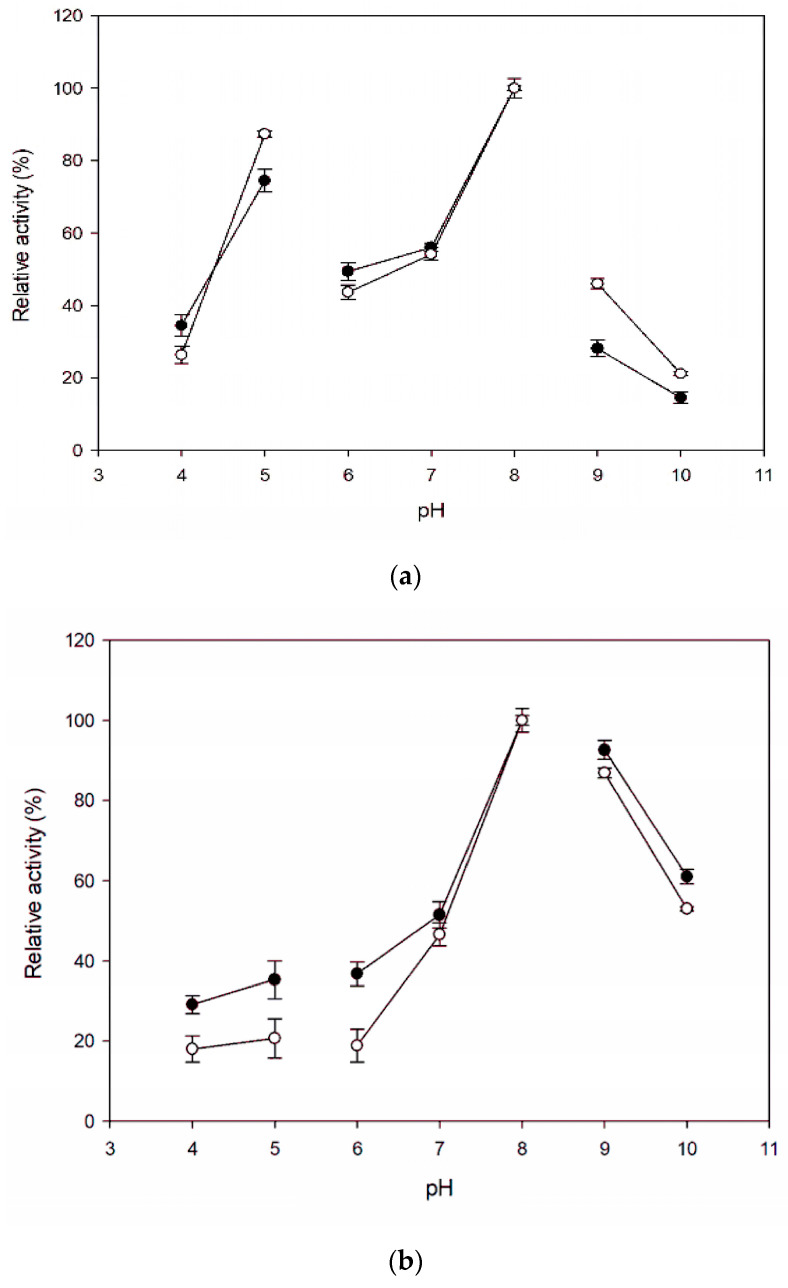
The effects of pH on (**a**) activity and (**b**) stability. The stability was examined by incubating the enzyme under a designated pH for 30 min before assaying its activity. Solid circle: TlCyn; open circle: TlCyn-SP. The activities of untreated TlCyn and TlCyn-SP were set as 100%.

Three different buffers at 20 mM were used to study the effect of pH: sodium acetate (pH 4–5), Tris-HCl (pH 6–8), and glycine-NaOH (pH 9–10). The effect of pH on activity is shown in Figure 3a. The optimal pH for both forms of TlCyn was 8, which is identical to the previously reported value [6]; the entrapment did not change the effect of pH on activity in the pH range of 4 to 8. The entrapment resulted in higher activity at pHs 9 and 10; a similar observation was reported for TlCyn immobilized on magnetic-multiwall carbon nanotubes [9]. The higher activity of TlCyn-SP is advantageous because cyanide-containing waste is usually alkaline [25], which is incompatible with pH-neutral enzymatic processes. The alkaline pH could change the electrostatic interactions and hydrogen bonds that are necessary for stabilizing the three-dimensional structure of the free enzyme, leading to lower activity. However, such conformational change is limited by the rigid silica network, which may explain the higher activity of the immobilized form. As for stability, both forms of TlCyn were stable at pHs 8 and 9 because at least 87% of the initial activities were retained (Figure 3b). However, the silica matrix did not improve the stability.

### 3.5. Cation Tolerance

**Figure 4:**
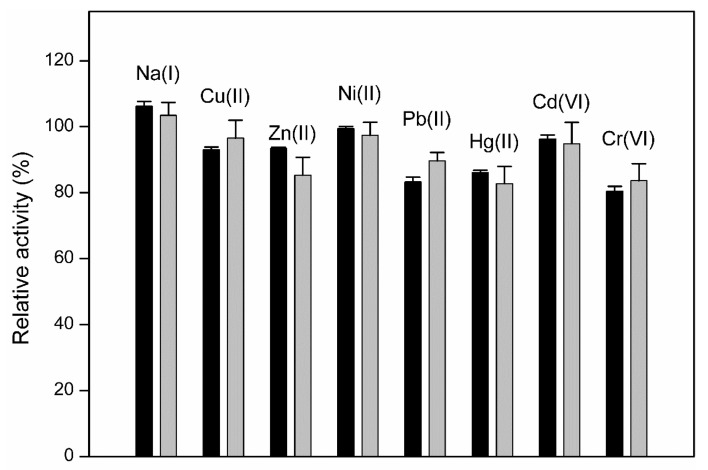
Metal ion tolerance. Black bar: TlCyn; gray bar: TlCyn-SP. The activities of untreated TlCyn and TlCyn-SP were set as 100%. The activity was assayed after 30 min of incubation with 5 mM metal ions.

Metal ions are often present in cyanide-containing wastewater. Therefore, the metal ion tolerance was examined, and the results are shown in Figure 4. Both forms of TlCyn tolerated the cations fairly well; at least 80% of the initial activities remained. There was little difference between the two forms of enzymes regarding the metal ion tolerance. Slight inhibition was observed for Cu(II), Zn(II), Ni(II), and Cd(II); somewhat stronger inhibition was observed for Pb(II), Hg(II), and Cr(VI), similar to a previous report for TlCyn [6].

### 3.6. Storage Stability

**Figure 5:**
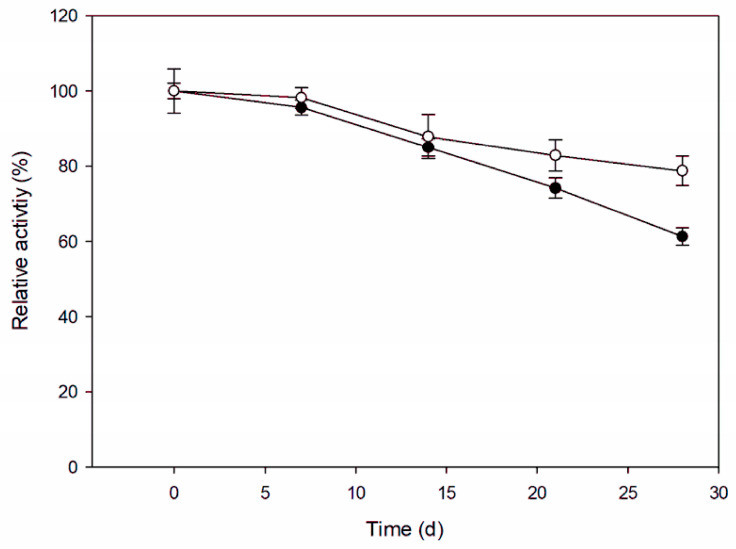
Storage stability at room temperature. Solid circle: TlCyn; open circle: TlCyn-SP. The initial activities were set as 100%.

Both TlCyn and TlCyn-SP retained 95% of their initial activities after 28 d of storage at 4 °C, suggesting that both forms of enzymes are stable at this temperature. The storage stability at 4 °C was similar to those of TlCyn immobilized on magnetic nanoparticles and magnetic multiwall carbon nanotubes [8,9]. The storage stability at room temperature is shown in Figure 5; the activity of the free enzyme decreased to 61% after 28 d, while the immobilized form retained 79% of the initial activity. The activity of the immobilized form showed a slower decrease at 25 °C in the latter half of the storage period, indicating that the silica entrapment improved the storage stability of TlCyn.

### 3.7. The Enzyme Kinetic Constants

The enzyme kinetic constants (Table 1) were derived from the Lineweaver–Burke plots. The free enzyme had a K_M_ of 0.44 mM, which is similar to the reported value [6]. The K_M_ of the entrapped enzyme decreased to 0.21 mM, indicating that the entrapped form has a stronger affinity towards cyanate. The entrapment increased the k_cat_ by 2.5-fold, from 18,417 to 45,881 s^−1^; such an increase was also observed for TlCyn immobilized on magnetic multiwall carbon nanotubes [9]. The increase in k_cat_ agrees with the enhanced activity of entrapped TlCyn described in Section 3.2, which can be explained by the stabilization of the enzyme structure at the assay temperature after entrapment. The entrapment increased the catalytic efficiency (k_cat_/K_M_) by 5.2-fold due to decreased K_M_ and increased k_cat_.

### 3.8. Cyanate Degradation Using TlCyn-SP and TlCyn-SazCA-SP

**Figure 6:**
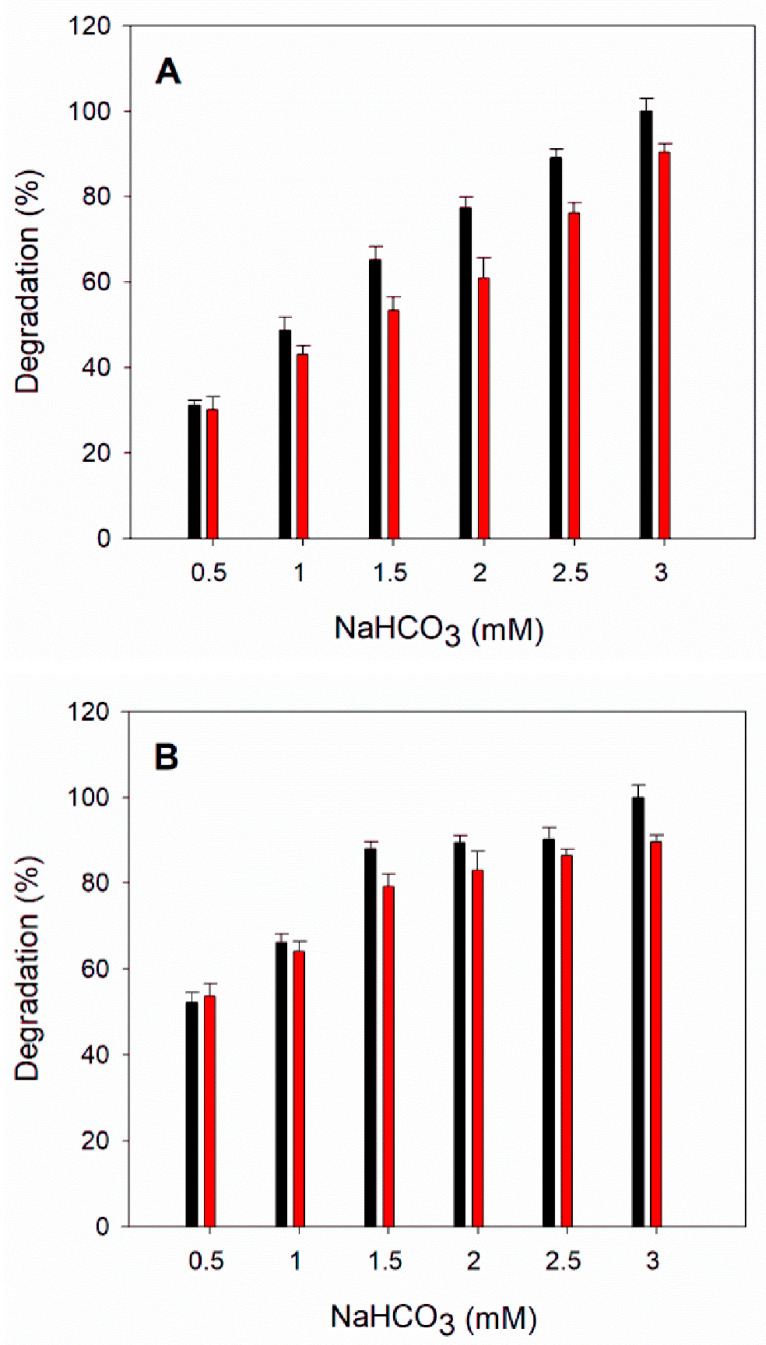
Degradation of cyanate in different matrices using entrapped enzymes. TlCyn-SP (**A**); TlCyn-SazCA-SP (**B**). The amounts of entrapped TlCyn and SazCA were both 300 ng. Degradation was performed in 50 mM Tris-HCl, pH 8 (black), and industrial wastewater (red). The 100% was defined as the cyanate degradation observed with 3 mM NaHCO_3_ in the buffer.

The cyanate degradation using only TlCyn-SP is shown in Figure 6A; the degradation increased with the HCO_3_^−^ concentration in both 50 mM Tris-HCl, pH 8, and industrial wastewater, showing that the degradation was dependent on HCO_3_^−^. The cyanate degradation in industrial wastewater was lower than that in the buffer, suggesting that the activity of the immobilized TlCyn was inhibited by the industrial wastewater. The industrial wastewater contained 2 mM Na(I), 0.2 µM Zn(II), 31.5 nM Cu(II), 44.3 nM Ni(II), 30.8 nM Cr(VI), and 4.8 nM Pb(II), as determined with ICP-OES. Based on the cation tolerance study in Section 3.5, Na(I) did not inhibit TlCyn, and the concentrations of Zn(II), Cu(II), Ni(II), Cr(VI), and Pb(II) were at least four orders of magnitude lower than the 5 mM used in the inhibition study. Therefore, the lower degradation observed in industrial wastewater most likely resulted from components other than metal ions.

**Scheme 1:**
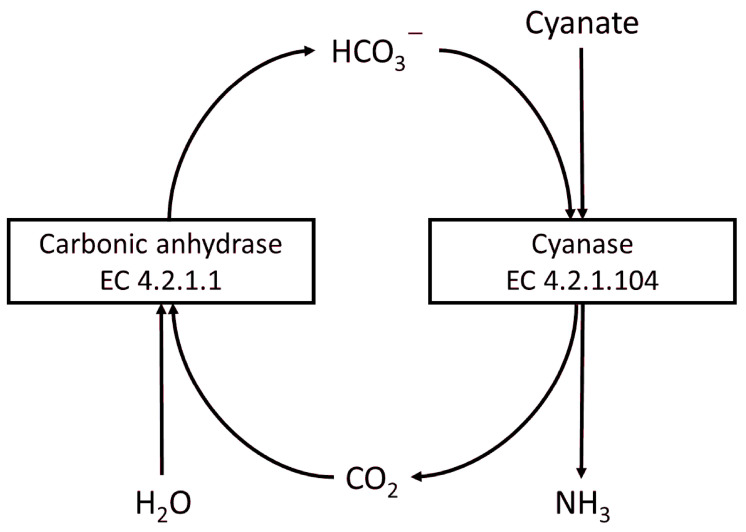
Catalyzed process of the interplay between cyanase and carbonic anhydrase.

The dependence on bicarbonate is a major hurdle for the large-scale application of cyanase; however, such dependence can be improved by coupling cyanase with carbonic anhydrase. Carbonic anhydrase catalyzes the hydration of CO_2_ (CO_2_ + H_2_O ⇌ HCO_3_^−^ + H^+^) [26], which provides the necessary bicarbonate for Cyn, and the consumed CO_2_ is replenished by Cyn. The catalyzed process of the interplay between cyanase and carbonic anhydrase is shown in the Scheme 1. The incorporation of carbonic anhydrase has been shown to decrease the required bicarbonate by 80% for cyanate degradation catalyzed by TlCyn [8].

The bicarbonate dependence was examined after the co-entrapment of carbonic anhydrase from *Sulfurihydrogenibium azorense* (SazCA) with TlCyn, and the results are shown in Figure 6B. SazCA was selected because of its good thermal stability and very high turnover rate (k_cat_ = 4.4 × 10^6^ s^−1^) [27]. The expression and purification of SazCA were described in our previous work [22]. In both matrices, the degradation increased with HCO_3_^−^ concentration between 0.5 and 1.5 mM NaHCO_3_; however, HCO_3_^−^ concentration had little effect on the degradation between 1.5 and 3 mM NaHCO_3_, showing that the bicarbonate dependence was overcome upon the co-entrapment of SazCA. For 50 mM Tris-HCl, pH 8, the cyanate degradation at 1.5 mM NaHCO_3_ was 35% lower than that at 3 mM NaHCO_3_ when only TlCyn-SP was used, and the difference was decreased to 12% when TlCyn-SazCA-SP was used. Similarly, the difference was decreased from 37% to 10% in industrial wastewater when TlCyn-SazCA-SP was used. The results clearly demonstrated the improvement in HCO_3_^−^ dependence by the co-entrapment of SazCA. Higher degradation was also achieved with TlCyn-SazCA-SP when compared with TlCyn-SP, which can be explained by the additional HCO_3_^−^ provided by SazCA.

### 3.9. Reusability of TlCyn-SP and TlCyn-SazCA-SP

**Figure 7:**
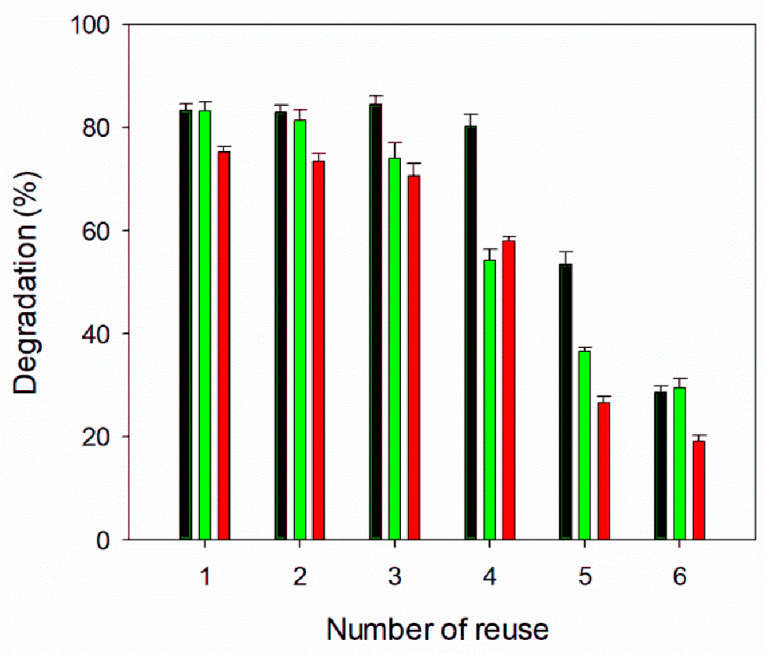
The reusability of entrapped enzymes for cyanate degradation. Degradation was performed with 3 mM NaHCO_3_ using TlCyn-SazCA-SP (dark green), with 1.5 mM NaHCO_3_ using TlCyn-SazCA-SP (light green) and with 3 mM NaHCO_3_ using TlCyn-SP (red). The amounts of entrapped TlCyn and SazCA were both 300 ng. Degradation was performed in 50 mM Tris-HCl, pH 8, and 100% was defined as the cyanate degradation before reuse.

The reusability of TlCyn-SP and TlCyn-SazCA-SP is shown in Figure 7. Three millimolar NaHCO_3_ was selected because the highest degradation can be obtained; however, 1.5 mM NaHCO_3_ was also used for TlCyn-SazCA-SP because the degradation was close to those obtained with 3 mM NaHCO_3_. TlCyn-SP retained 58% of the initial degradation after four cycles of reuse; however, the degradation decreased significantly in the fifth and sixth cycles, with 26% and 19% of the initial degradation remaining, respectively. For TlCyn-SazCA-SP at 1.5 mM NaHCO_3_, the reusability was similar to that of TlCyn-SP, with 54% of the initial degradation remaining after four cycles. The reusability was improved for TlCyn-SazCA-SP when 3 mM NaHCO_3_ was used; the degradation was very similar for the first four cycles, and 53% of the initial degradation was retained, even after the fifth cycle. It has been shown that SazCA entrapped within biomimetic silica retained most of its activity after 180 min of incubation at 60 °C [22], the temperature used to examine the degradation. Therefore, the decrease in degradation during reuse is likely due to the inactivation of TlCyn-SP at 60 °C (discussed in Section 3.3). The loss of silica particles due to washing and surface adsorption may also contribute to the decrease in degradation.

## 4. Conclusions

The entrapment of TlCyn within biomimetic silica significantly improves the enzyme’s thermal stability, storage stability, and reusability. The entrapment process is rapid and simple; it does not require prior support-activation. Entrapped TlCyn is successfully applied to cyanate degradation, and the bicarbonate dependence of the reaction is alleviated by simultaneously entrapping SazCA. The entrapped enzymes can be reused up to five times while still maintaining over 50% cyanate degradation efficiency; the reusability should significantly lower the cost of large-scale applications that require these enzymes. Entrapped TlCyn can serve as a robust biocatalyst for other applications besides cyanate degradation.

## Figures and Tables

**Table 1:** The kinetic constants of TlCyn and TlCyn-SP.

Samples	K_M_ (mM)	k_cat_ (s^−1^)	k_cat_/K_M_ (s^−1^·mM^−1^)
TlCyn	0.44	18,417	41,857
TlCyn-SP	0.21	45,881	218,481

## Data Availability

Data can be provided by the authors upon request.
